# The Myc/Max/Mxd Network Is a Target of Mutated Flt3 Signaling in Hematopoietic Stem Cells in Flt3-ITD-Induced Myeloproliferative Disease

**DOI:** 10.1155/2018/3286949

**Published:** 2018-10-21

**Authors:** Farhan Basit, Maria Andersson, Anne Hultquist

**Affiliations:** ^1^Hematopoietic Stem Cell Laboratory, Lund Stem Cell Center, Lund University, Klinikgatan 26, 221 84 Lund, Sweden; ^2^Department of Tumor Immunology, Radboud Institute for Molecular Life Sciences, Radboudumc, Nijmegen, Netherlands

## Abstract

Acute myeloid leukemia (AML) has poor prognosis due to various mutations, e.g., in the FLT3 gene. Therefore, it is important to identify pathways regulated by the activated Flt3 receptor for the discovery of new therapeutic targets. The Myc network of oncogenes and tumor suppressor genes is involved in mechanisms regulating proliferation and survival of cells, including that of the hematopoietic system. In this study, we evaluated the expression of the *Myc* oncogenes and *Mxd* antagonists in hematopoietic stem cell and myeloid progenitor populations in the Flt3-ITD-knockin myeloproliferative mouse model. Our data shows that the expression of Myc network genes is changed in Flt3-ITD mice compared with the wild type. *Mycn* is increased in multipotent progenitors and in the pre-GM compartment of myeloid progenitors in the ITD mice while the expression of several genes in the tumor suppressor *Mxd* family, including *Mxd1*, *Mxd2*, and *Mxd4*, is concomitantly downregulated, as well as the expression of the Mxd-related gene *Mnt* and the transcriptional activator *Miz-1*. LSKCD150^+^CD48^−^ hematopoietic long-term stem cells are decreased in the Flt3-ITD cells while multipotent progenitors are increased. Of note, PKC412-mediated inhibition of Flt3-ITD signaling results in downregulation of *cMyc* and upregulation of the Myc antagonists *Mxd1*, *Mxd2*, and *Mxd4*. Our data provides new mechanistic insights into downstream alterations upon aberrant Flt3 signaling and rationale for combination therapies for tyrosine kinase inhibitors with Myc antagonists in treating AML.

## 1. Introduction

Acute myeloid leukemia (AML), despite aggressive treatment regimes, has a poor prognosis, and cure is difficult to attain. Mutations in the tyrosine kinase receptor Flt3, including internal tandem duplication (ITD) mutations and tyrosine kinase domain (TKD) mutations, are the most common changes in AML, which constitutively activate the Flt3 receptor [1]. Flt3 regulates growth and survival of myeloid progenitor cells; therefore, mutations in *FLT3* effectively abrogate growth-regulating controls [2]. Interestingly, mutations in *FLT3* are correlated with shorter progression-free survival and overall survival [3, 4].

Treatment strategies aimed at inhibiting the activated Flt3-ITD receptor have been evaluated in clinical trials; however, the use of Flt3-ITD inhibitors as single agents resulted in poor clinical outcome due to emergence of drug-resistant cells [5]. This underscores the need to develop combination treatment strategies [6]. Therefore, it is important to identify pathways regulated by the activated Flt3 receptor for the development of new treatment targets. Several pathways have been implicated downstream of the mutated Flt3 receptor in leukemias, including the Wnt pathway and the JAK/STAT pathway [7, 8].

Interestingly, the *MYC* oncogenes have been implicated downstream of Flt3-ITD signaling [9]. The *MYC* family genes, including *MYC*, *MYCN*, and *MYCL1*, are proto-oncogenes and known to be overexpressed or mutated in a plethora of different tumors, including that of the hematopoietic system [10, 11]. The MYC proteins function as transcription factors and bind to specific E-box DNA sequences, in promoters of target genes by heterodimerizing with their partner MAX [12]. However, Myc also regulates genes independent of DNA binding via Miz-1 protein [13]. Intriguingly, bone marrow-specific overexpression of Mycn results in rapid development of acute myeloid leukemia [14]. Furthermore, Myc has also been shown to induce myeloid myeloproliferative disease, even though mutations in myeloid neoplasias are not common [15]. The Mxd family of proteins also heterodimerizes with Max and binds to the common E-box sequences and generally works in an antagonistic way towards Myc [12].

Importantly, several reports have implicated Myc as a downstream target of Flt3-ITD signaling. To this end, it was shown that Flt3-ITD regulates cMyc via Wnt signaling [8]. Additionally, Flt3-ITD inhibits Foxo3a [16], which in turn suppresses the Myc antagonist Mxi-1 (Mxd2) to increase Myc activity [17]. Interestingly, studies of Myc function in hematopoietic stem cells have implicated Myc in pushing the HSCs out of the niche and into a proliferative progenitor state [18]. Given that both Flt3 and Myc regulate HSCs' self-renewal and differentiation, evaluating the interplay between Flt3-ITD signaling and Myc molecules may represent therapeutic targets for AML therapy.

In this study, we investigated the Myc network genes in different subpopulations in the bone marrow, including myeloid progenitors and stem cell populations, in the Flt3-ITD myeloproliferative mouse model [19]. Here, we report that the expression of the Myc network genes is changed in the Flt3-ITD mouse model, mainly with upregulation of the *Myc* genes and concomitant downregulation of the Myc antagonists, the *Mxd* genes, in different hematopoietic stem and progenitor cell subpopulations, as well as downregulation of the Mxd-related gene *Mnt* and the transcriptional activator *Miz-1*. Moreover, PKC412-mediated inhibition of Flt3-ITD signaling results in downregulation of *c-Myc* and upregulation of the Myc antagonists *Mxd1*, *Mxd2/Mxi1*, and *Mxd4*.

## 2. Materials and Methods

### 2.1. Cell Culture and PKC412 Inhibitor

MV4-11 cells were cultured in IMDM medium (Sigma-Aldrich) supplemented with 20% FCS, 1% L-glutamine, and 1% penicillin/streptomycin. Cells were constantly maintained at 37°C in 5% CO_2_. The Flt3-ITD phosphokinase inhibitor was added to cell cultures of MV4-11 cells in 2 different concentrations for 15 minutes before the cells were harvested for mRNA extraction (0.1 mM and 1 mM) (LC Laboratories, Woburn, MA, USA).

### 2.2. Mice

Flt3-ITD-knockin mice on C57BL/6 background were previously described [19]. WT (Flt3^+/+^) C57BL/6 littermate mice were used as WT controls. All experiments were approved by the Ethical Committee at Lund University.

### 2.3. Fluorescent Antibodies and Immunomagnetic Beads Used for FACS Analysis and Sorting

Antibodies used for cell surface staining were as follows: CD11b/Mac1 (M1/70), CD4 (H129.19), CD8a (53–6.7), B220/CD45 (RA3–6B2), CD5 (Ly1), Ter119 (Ter-1119), Gr1/Ly6G and Ly6C (RB6–8C5), CD19 (ID3), CD41/Itga2b (MWReg30), and CD135/Flt3 (A2F10.1) (104) (BD Biosciences Pharmingen) and NK1.1 (PK136), Sca1 (D7), CD117/c-Kit (2B8), CD16/32 (93), and CD105/Eng (MJ7/18) (eBioscience). Biotinylated antibodies were visualized with streptavidin-QD655 (Invitrogen) or streptavidin-tricolor (Invitrogen), and purified lineage antibodies were visualized with polyclonal goat anti-rat tricolor (Invitrogen) or polyclonal goat anti-rat-QD605 (Invitrogen). MACS column enrichment of c-Kit^+^ cells was done using anti-CD117 immunomagnetic beads (Miltenyi Biotec) as previously described [20].

### 2.4. Flow Cytometric Analysis

Hematopoietic stem and progenitor cells were analyzed as previously described. [21–23]. Briefly, bone marrow (BM) cells were stained with a cocktail of purified rat antibodies against lineage markers B220, CD4, CD5, CD8*α*, CD11b, Gr1, and Ter119. Lineage^+^ cells were visualized with a goat anti-rat-QD605 staining, followed by c-Kit enrichment for sorting analyses. Thereafter, hematopoietic stem/progenitor cells were defined as Lin^−^Sca1^+^c-Kit^+^ (LSK) cells, pre-granulocyte-monocyte progenitors (pre-GMPs: Lin^−^c^−^Kit^+^Sca1^−^ [LSK^−^] CD41^−^CD16/32^low/−^CD150^−^CD105^low/−^), granulocyte-monocyte progenitors (GMPs: LKS^−^CD16/32^hi^CD150^−^), and multipotent progenitors (MPPs: Lin^−^Sca1^+^ c-Kit^+^CD150^−^CD105^low/−)^. Propidium iodide (Invitrogen) was used to exclude dead cells. Cell acquisition and analysis were performed on a 4-laser LSRII (BD Biosciences) using FlowJo version 8.8 software (TreeStar). Cell sorting was done on a FACSAria (BD Biosciences).

### 2.5. Quantitative Real-Time PCR

For analyzing gene expression in myeloid progenitors (pre-GM, GMPs) as well as in hematopoietic stem cells and multipotent progenitors (MPPs), cells from these populations were FACSAria-sorted directly into 75 *μ*l of buffer RLT and frozen at −80°C. Total RNA extraction and DNase treatment were performed with the RNeasy Micro kit (Qiagen Inc., California) according to the manufacturer's instructions for samples containing ≤10^5^ cells. Eluted RNA samples were reverse-transcribed using the SuperScript II Reverse Transcriptase Kit including random hexamers (Invitrogen) according to the protocol supplied by the manufacturer. For gene expression in LT-HSCs as well as in MPPs using the Slam marker staining (including CD150 and CD48), the CelluLyser™ protocol (TaTaa, Gothenburg, Sweden) was used according to the manufacturer's protocol. Shortly, cells were FACSAria-sorted into 5.5 ∝ l lysis buffer where RNA was directly reverse-transcribed using the Transcriptor First-Strand cDNA synthesis kit (Roche). Q-PCR reactions with the diluted cDNA samples were analyzed with TaqMan gene expression assays (ABI, USA) according to the manufacturer's protocol. TaqMan Assays-on-Demand probes used are described in the Supplemental Experimental Procedures. All experiments were performed in triplicate and from at least two different sorts, and differences in cDNA input were compensated by normalizing against *β*-actin or HPRT expression levels. The fold induction ratio was calculated by the Pfaffl equation:

(1) $$\begin{array}{c} ratio=\frac{\left(E_{target}\right)\Delta Ct\;target\left(control-sample\right)}{\left(E_{ref}\right)\Delta Ct\;ref\left(control-sample\right)}. \end{array}$$

Statistical analysis was performed with two-tailed unpaired Student's *t*-test on log-converted values (^∗^*p* < 0.05, ^∗∗^*p* < 0.01).

## 3. Results

### 3.1. Myeloid Progenitor and Hematopoietic Stem Cell Populations Are Changed in Flt3-ITD Mice

To evaluate the gene expression of the *MYC* network genes in the bone marrow of Flt3-ITD mice compared with wild-type (WT) mice, hematopoietic stem cell and myeloid progenitor (MPP) subpopulations were identified by staining for surface markers, analyzed by fluorescence-activated cell sorting (FACS), and subsequently sorted. Initially, myeloid progenitors including pre-GM and granulocytic myeloid progenitors (GMPs), as well as Lin^−^Sca-1^+^Kit^+^ (LSK) cells, within which hematopoietic stem cells reside, were sorted using a staining procedure including endoglin and the SLAM receptor CD150, as described previously [23].

**Figure 1:**
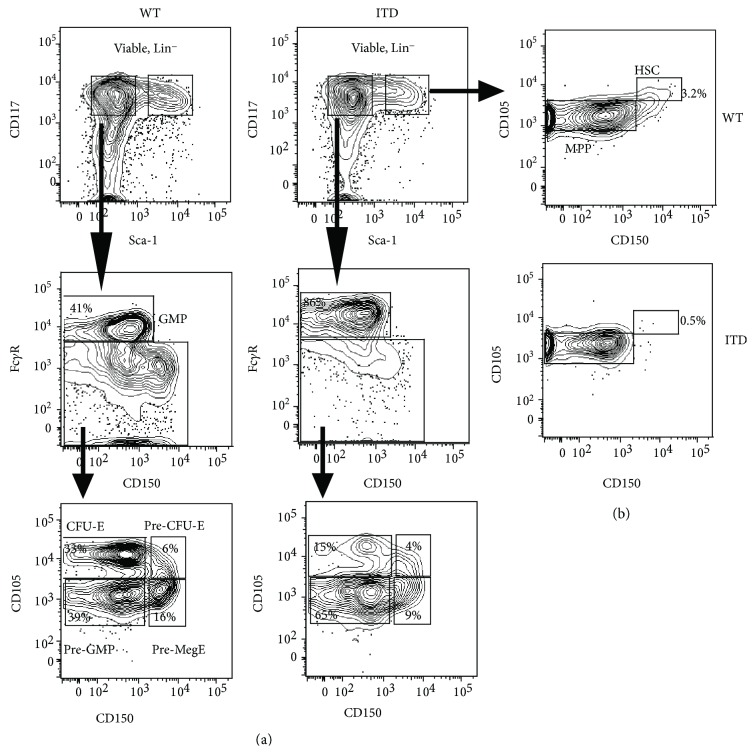
Myeloid progenitor and hematopoietic stem cell populations are changed in Flt3-ITD mice. Analysis of hematopoietic stem cell (HSC) and multipotent progenitor (MPP) subpopulations within the Lin^−^Sca-1^+^Kit^+^ (LSK) population, as well as myeloid progenitors including pre-GM and granulocytic myeloid progenitors (GMPs), in the bone marrow of Flt3-ITD and wild-type (WT) mice, was performed using a staining procedure including endoglin and the SLAM receptor CD150.

The Flt3-ITD mouse has a myeloproliferative disease with expanded myeloid populations [19]. Consistently, we observed that myeloid progenitors (MPs/LK; Lin^−^Sca-1^−^Kit^+^ cells) in Flt3-ITD mice are increased to 69.5% in comparison with 54.9% MPs in WT mice (Figure 1(a)). Moreover, we observed that the relative distribution of subpopulations within the MP compartment was altered as well. Importantly, progenitors of the granulocytic/monocytic pathway (pre-GM and GMPs) were increased in Flt3-ITD mice in comparison to WT mice, as we observed that pre-GMs were increased from 39% in WT to 65% in Flt3-ITD mice and GMPs were increased from 41% in WT to 86% in ITD mice (Figure 1(a)). Consistent with our previous data [24], the progenitors of the megakaryocytic and erythroid pathway were diminished (Figure 1(a)). The expression of Flt3 is altered or diminished due to the ITD mutation; therefore, staining to identify HSC subpopulations by utilizing the expression of the Flt3 receptor is not feasible. Here, we identified long-term (LT-) HSCs as CD150^+^CD105^+^ utilizing CD150 and endoglin (CD105), while MPPs were identified as CD150^−^/CD105^+^. Intriguingly, we observed a decrease in LT-HSCs in favor of MPPs in the ITD mice (Figure 1(b)). Collectively, the above data set indicates that ITD mutation in Flt3 results in the expansion of the pre-GM, GMP, and MPP compartments.

### 3.2. Myeloid and Multipotent Progenitors Have Altered Myc Network Genes in Flt3-ITD Mice

**Figure 2:**
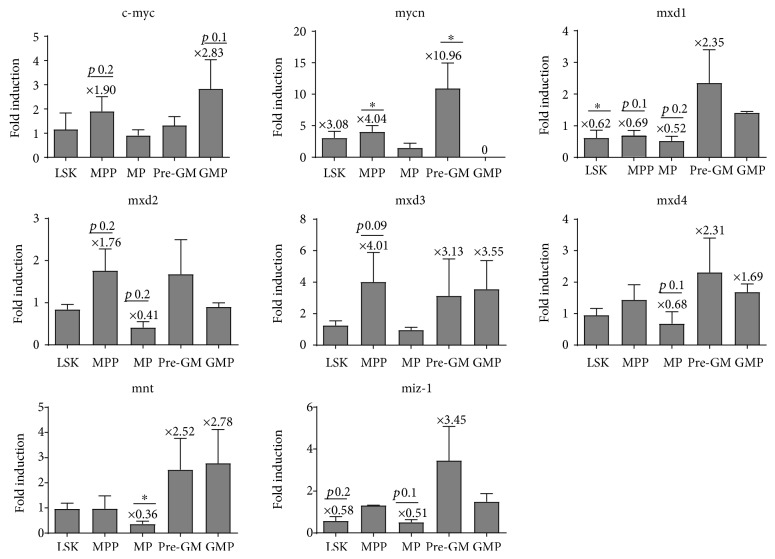
Myeloid and multipotent progenitors have altered Myc network genes in Flt3-ITD mice. The expression of Myc network genes was carried out with reverse transcription quantitative PCR (RT-Q-PCR) in sorted subpopulations of murine hematopoietic stem and progenitor cells. Fold induction was calculated as a ratio of Flt3-ITD : WT samples. Statistical analysis was performed with Student's *t*-test on log-converted values (^∗^*p* < 0.05). Significance was shown in many of the described altered expression levels; however, some changes showed low *p* values but did not quite reach statistically significance due to variances in the expression levels.

Next, we evaluated the expression of the *Myc* network genes including *cMyc* and *Mycn*, as well as the *Mxd* family of Myc antagonists (*Mxd1*, *Mxd2/Mxi1*, *Mxd3*, and *Mxd4*), in stem and progenitor subpopulations in mice with the Flt3-ITD mutation and littermate WT controls by quantitative real-time PCR (Q-RT-PCR) (Figure 2). The mRNA of the *cMyc* gene was increased in Flt3-ITD MPPs (1.9-fold induction), as well as in Flt3-ITD GMPs (2.83-fold induction). Of note, the *Mycn* expression was increased in all the populations investigated in Flt3-ITD mice, except for GMPs where the expression of *Mycn* was turned off. *Mycn* was most prominently upregulated in MPPs and pre-GM cells (4.04- and 10.96-fold induction, respectively). Furthermore, analysis of the expression of the Myc antagonists, the *Mad* family genes, showed downregulation of *Mxd1* in LSK cells (0.62-fold reduction), MPPs (0.69-fold reduction), and MPs (0.52-fold reduction). *Mxd2/Mxi1* was downregulated in LSK (0.77-fold reduction) and MP (0.41-fold reduction) cells and *Mxd4* in MPs (0.68-fold reduction). Additionally, *Mxd2*/*Mxi1* and *Mxd3* were upregulated in MPPs. The *MNT* gene, an *MXD* family-related gene, which is coexpressed with Myc in proliferating cells and functions as a repressor of Myc target genes [25] was downregulated (0.36-fold reduction) in myeloid progenitors (MPs) in Flt3-ITD mice. Similarly, the *MIZ-1* gene, a transcriptional activator which is involved in upregulating growth-repressing genes such as p21, was downregulated in LSK cells (0.58-fold reduction) and MPs (0.51-fold reduction). *Max*, the Myc- and Mxd-interacting partner, was significantly increased in MPPs (1.75-fold induction) and significantly decreased in MPs (0.48-fold reduction) (data not shown). Collectively, these data indicate that the ITD mutation in Flt3 results in the alteration of Myc network genes.

### 3.3. Hematopoietic Stem Cells in Flt3-ITD Mice Have Altered Myc Network Expression

**Figure 3:**
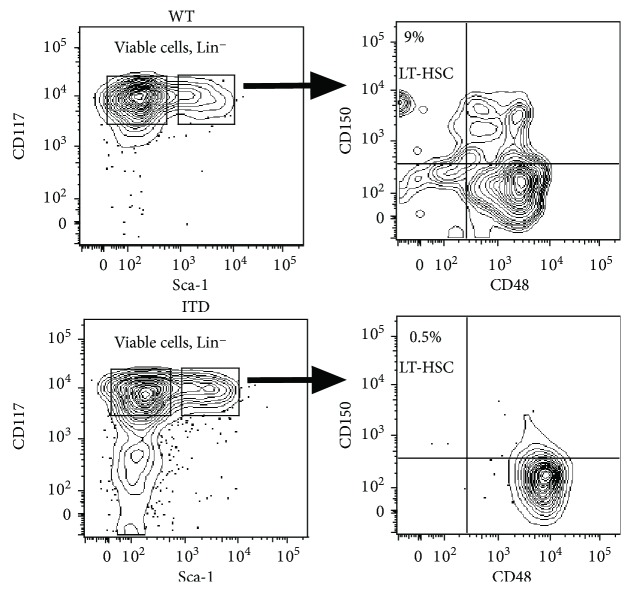
Flt3-ITD mice have altered frequencies of hematopoietic stem and progenitor cells. Next, we subdivided the hematopoietic stem cell compartment into long-term HSCs (LT-HSCs) and MPPs, utilizing the SLAM receptor staining with CD150 and CD48, and cells were sorted by flow cytometry for subsequent gene expression analysis.

Next, we subdivided the hematopoietic stem cell compartment into long-term HSCs (LT-HSCs) and MPPs, utilizing the SLAM receptor staining with CD150 and CD48 [22], and cells were sorted by flow cytometry for subsequent gene expression analysis. Of note, the LT-HSCs (LSKCD150^+^CD48^−^) were decreased in the Flt3-ITD cells, as described previously [26], and the MPPs were increased. Intriguingly, MPPs expressed higher levels of CD48 (Figure 3).

**Figure 4:**
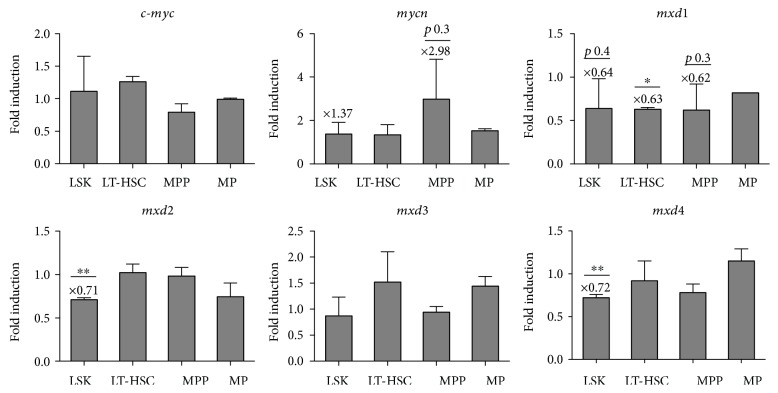
Hematopoietic stem cells in Flt3-ITD mice have altered Myc network expression. The expression of Myc network genes was carried out with reverse transcription quantitative PCR (RT-Q-PCR) in hematopoietic stem cells. Fold induction was calculated as a ratio of Flt3-ITD : WT samples. Statistical analysis was performed with Student's *t*-test on log-converted values (^∗^*p* < 0.05, ^∗∗^*p* < 0.01).

Expression of the *Myc* network genes was evaluated in LSK, LT-HSC (CD150^+^CD48^−^), MPP (CD150^−^CD48^+^), and MP cells. Intriguingly, the expression of the *cMyc* gene did not change in LSK, LT-HSC, MPP, and MP cells as identified in the SLAM receptor-based staining in contrast to the staining including the endoglin marker (Figures 4 and 1). Conversely, the *Mycn* mRNA was increased in MPP cells (2.98-fold induction) (Figure 4), comparative with the results from the analysis in the endoglin/CD150 staining protocol (Figure 2). However, the results in this staining did not quite reach significant values due to variations. Importantly, *Mxd1* showed a significant decrease in expression in LT-HSCs (0.63-fold reduction), as well as in MPPs (0.62-fold reduction) and the LSK compartment (0.64-fold reduction), which, however, did not reach significant levels (Figure 4). Of note, *Mxd2/Mxi1* and *Mxd4* were significantly decreased in the LSK compartment; however, their expression did not change in LT-HSCs, MPPs, and MPs. Conversely, *Mxd3* did not change significantly in any of the subpopulations (Figure 4).

### 3.4. Small Molecule Inhibitor, PKC412, Modulates the Expression of *Myc* Network Genes in Human MV4-11 Cells

**Figure 5:**
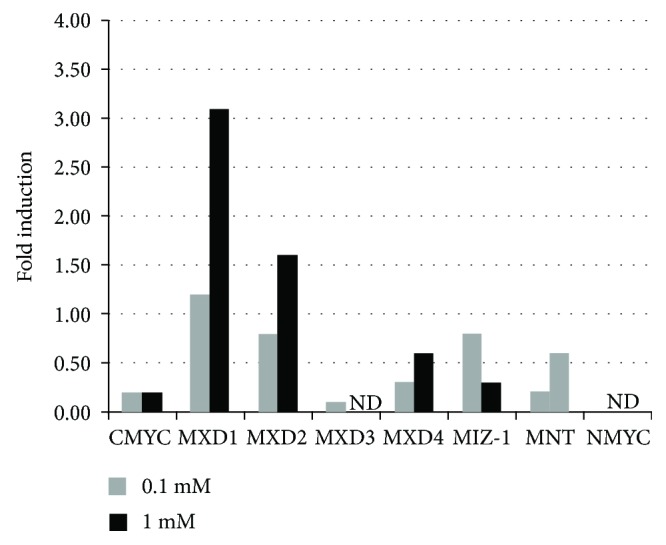
Small molecule inhibitor, PKC412, modulates the expression of *Myc* network genes in human MV4-11 cells. The human Flt3-ITD mutated leukemia cell line, MV4-11, was treated with the Flt3-inhibitor PKC412 at two different concentrations (0.1 mM and 1 mM) for 15 minutes. Expression analysis of the Myc network genes was carried out with RT-Q-PCR. ND: not detected.

PKC412 is an inhibitor of FLT3 autophosphorylation, thereby inhibiting downstream signaling [27]. It has shown to inhibit growth of primary Flt3-ITD mutant blasts [28]. To investigate whether Flt3-ITD inhibition exerts antileukemic activity via modulation of the Myc network, we treated the human Flt3-ITD mutated leukemia cell line, MV4-11, with PKC412 at two different concentrations (0.1 mM and 1 mM) and analyzed the expression of Myc network genes. Of note, PKC412 reduced *cMyc* expression to the same extent at both concentrations. Conversely, the expression of the Myc antagonists *Mxd1* and *Mxd2* was increased in a dose-dependent manner in MV4-11 cells treated with PKC412 (Figure 5), as well as the expression of *Mxd4*, but to a lesser extent. Intriguingly, MV4-11 cells do not express *Mycn* (Figure 5).

## 4. Discussion

Currently, a rapid development of targeted therapies against specifically overexpressed or mutated molecules in AML is ongoing. Clinical trials against mutated molecules found in AML, e.g., IDH1 and IDH2, have recently been initiated [29]. Considering the risk of relapse with the current treatment, it is important to identify pathways regulated by the activated Flt3 receptor for the identification of new treatment targets. The Myc network of oncogenes and tumor suppressors is often changed in a plethora of tumors. The overexpression of the *MYC* and *MYCN* oncogenes is mostly not due to actual mutations in the genes, but their expression is deregulated due to upstream activated pathways and molecules [30]. However, there are exceptions to the rule, as *MYCN* is amplified in neuroblastomas [31]. *Myc* and *Mycn* overexpression has been shown to initiate myeloid and lymphoid neoplasms and could therefore be possible targets for inhibiting leukemic cell proliferation and viability. The Mxd network of tumor suppressors has been shown to be downregulated or deleted in different tumor types including prostate adenocarcinoma [32]. In this study, we have analyzed the possible role of the Myc network in Flt3-ITD-induced myeloproliferative disease. Flt3-ITD is one of the most common mutations in AML and is correlated with poor prognosis. Strategies of inhibiting the overactivated Flt3-ITD tyrosine kinase or pathways downstream of this receptor would therefore be of interest in treating AML patients with this mutation. Efforts with Flt3-ITD inhibitors to treat AML are ongoing. However, results have shown that the Flt3-ITD inhibitor should be used in combination with other treatments to avoid the development of drug resistance.

Herein, we report that myeloid progenitors (MPs) are increased in adult Flt-3ITD mice, which is consistent with other reports. Intriguingly, fetal Flt3-ITD mice have a normal MP compartment and are protected from leukemic transformation [33]. Similarly, LT-HSCs (LSKCD150^+^48^−^) were reported to be present in normal numbers in Flt3-ITD fetal livers before the onset of myeloproliferative disease. However, we here show that LT-HSCs decrease in favor of MPPs in Flt3-ITD mice which has developed a myeloproliferative disease, which has also been shown in other reports ([34, 35, 26]). Evidence has shown that the effect of Flt3-ITD on LT-HSC homeostasis is cell autonomous [26]. Changes in the expression of the *Myc* network genes downstream of Flt3-ITD could therefore be responsible for an expansion of leukemic multipotent progenitors. Of note, STAT3 is upregulated in Flt3-ITD MPPs [34]. Moreover, in adult progenitors, Flt3-ITD induces self-renewal in a STAT5-dependent manner [36]. Interestingly, lineage-specific STAT5 activation in hematopoietic progenitor cells predicts the FLT3^+^-mediated leukemic phenotype in mice [37], and the STAT signaling pathway has been reported to increase Myc activity. These data highlight the involvement of STAT signaling in connection with the Myc network in aberrant hematopoietic stem and progenitor cell populations in Flt3-ITD, which is thus also a potential therapeutic target in Flt3-ITD leukemia.

We found that the Myc network of oncogenes and tumor suppressors is changed in Flt3-ITD myeloproliferative mice compared with wild-type mice. Generally, the *Myc* gene expression was increased, and the expression of the Myc antagonists, mainly *Mxd1*, *Mxd2/Mxi1*, *Mxd4*, *Mnt*, and *Miz-1*, was decreased. Myc has been shown to be involved in displacing quiescent hematopoietic stem cells from their niche to more proliferative progenitor cells [38]. This can be correlated with our results showing the change in hematopoietic stem and progenitor cell subpopulation distribution, where the long-term hematopoietic stem cells are decreased in Flt3-ITD mice and the multipotent progenitors increased. Our data shows that *cMyc* was increased in Flt3-ITD multipotent progenitors (MPPs) as was *Mycn* in LSK, MPP, and pre-GM cells. Importantly, c-MYC has been reported to induce the expression of the deubiquitinase USP22, which in turn reduced ubiquitination and enhanced the stability of SIRT1 in CD34^+^ Flt3-ITD cells. Of note, inhibition of SIRT1 expression or activity reduced the growth of Flt3-ITD AML [39]. Additionally, c-MYC generates repair errors by regulating transcriptional activation and expression of the alternative nonhomologous end-joining pathway resulting in aberrant DNA repair in Flt3-ITD leukemia [40]. Further, N-Myc overexpression mechanistically results in the hyperproliferation of myeloid cells by decreasing transforming growth factor *β* signaling and increasing c-Jun-NH2-kinase signaling to cause AML [14]. Collectively, these data underscore the importance of inhibition of the Myc molecules in treating Flt3-ITD mutated AML. Furthermore, we observed the downregulation of *Mxd* family genes, i.e., *Mxd1*, *Mxd2/Mxi1*, and *Mxd4*. Interestingly, Krüppel-like factor 4 (KLF4) has been identified as an upstream transcriptional regulator of *Mxd1* and *Myc* in myeloid leukemias [41]. Intriguingly, while SIRT1 was shown to regulate c-MYC in Flt3-ITD mutated leukemia [39], it has been demonstrated to regulate *Mxd1* in malignant melanoma [42]. Similarly, we found that *Mnt* and *Miz-1* were also downregulated in Flt3-ITD MPs. Of note, *Miz-1* serves as a platform to inhibit the expression of cell cycle regulators ([43, 44]). Flt3-ITD mutated leukemic cells have enhanced activity of Cdc25, which overrides the replication checkpoint leading to arrest in the S phase [45]. These findings could point to the possibility that reduced levels of Miz-1 result in enhanced activity of Cdc25 thereby deregulating the cell cycle in Flt3-ITD leukemia. Given that compromised DNA damage response and weakened cell cycle checkpoint promote the progression of AML, our data points to the potential role of *Myc* and *Miz-1* in regulating these pathways in Flt3-ITD leukemia.

As the phosphorylation status of FLT3 is associated with its functional activity [46], the inhibition of FLT3 phosphorylation will affect FLT3-dependent pathways such as RAS/MAPK, JAK/STAT, and Wnt pathways [9]. Given that Myc oncogenes are downstream effectors of these pathways and based on our results that *Myc* oncogenes are altered upon ITD mutation in *FLT3*, we hypothesized that modulation of FLT3 phosphorylation will result in transcriptional reprogramming of the Myc network. Our data showed that PKC412-mediated inhibition of FLT3 signaling increased *Mxd1* and *Mxd2* expression, as well as the expression of *Mxd4* and *Mnt* to a lesser degree, while it reduced *cMyc* expression in Flt3-ITD AML cells. During the course of preparation of this manuscript, Zhang et al. reported that PKC412-induced Myc downregulation results in decreased telomerase reverse transcriptase (hTERT) activity [47]. Additionally, inhibition of cMyc has several therapeutic implications in solid tumors and hematological malignancies. It has been reported that cMyc inhibition overcomes radio- and chemotherapy resistance in pediatric medulloblastoma [48]. Similarly, cMyc inhibition has been shown to negatively impact lymphoma growth [49] and overcome drug-resistant AML [33]. Furthermore, cMyc inhibition prevents leukemia initiation in mice and impairs the growth of relapsed and induction failure pediatric T-ALL cells [50]. Our data showed that selective inhibition of Flt3-ITD downstream signaling induced c-Myc inhibition, which is consistent with a recent report [47]. Furthermore, our data also shows that *Mnt* and *Miz-1* of the *Mxd* family are targets of Flt3-ITD signaling pointing to the Myc network as a whole being a target of activated Flt3-ITD. Additionally, our data showed that *nMyc* expression is increased in LSK, MPP, and pre-GM cells from Flt3-ITD mice. Reports supporting the important role of the inhibition of the Mxd family of tumor suppressors include studies showing that Mxd1 promotes cell cycle arrest and differentiation [30]; also, several studies showed deletion of the 10q24-q25 chromosome, where the *MXD2* gene is located in solid tumors [51]. Furthermore, *MXD2* is mutated in hematological malignancies [52], as well as in solid tumors. Interestingly, reintroduction of Mxd2 in glioblastoma cells deficient in Mxd2 results in reduced glioblastoma cell growth and clonogenicity [53]. Our study points to the fact that alterations in the Myc network by Flt3-ITD signaling are involved in myeloid leukemogenesis and that PKC412-mediated Flt3-ITD inhibition partly exerts its antileukemic activity by affecting the Myc/Max/Mxd network.

## Data Availability

The Q-RT-PCR data and flow cytometry data used to support the findings of this study are included within the article.
